# Preventing food allergy: protocol for a rapid systematic review

**DOI:** 10.1186/2045-7022-3-10

**Published:** 2013-03-28

**Authors:** Debra de Silva, Sukhmeet S Panesar, Sundeep Thusu, Tamara Rader, Susanne Halken, Antonella Muraro, Aziz Sheikh

**Affiliations:** 1 The Evidence Centre, 126 Central Avenue, London, England, TW3 2RJ, UK; 2 University of Edinburgh, Teviot Place, Edinburgh, EH8 9AG, UK; 3 Centre for Transcultural Oral Health, King’s College London, Strand, London, WC2R 2LS, UK; 4 University of Ottawa, 75 Laurier Avenue East, Ottawa, ON, K1N 6N5, Canada; 5 Odense University Hospital, Sdr. Boulevard 29, 5000, Odense C, Denmark; 6 Padua General University Hospital, Via Giustiniani 3, Padua, 35128, Italy

**Keywords:** Food allergy, lLgE-mediated, Prevention

## Abstract

**Background:**

The European Academy of Allergy and Clinical Immunology is developing guidelines about how to prevent and manage food allergy. As part of the guidelines development process, a systematic review is planned to examine published research about the prevention of food allergy. This systematic review is one of seven inter-linked evidence syntheses that are being undertaken in order to provide a state-of-the-art synopsis of the current evidence base in relation to epidemiology, prevention, diagnosis and clinical management, and impact on quality of life, which will be used to inform clinical recommendations. The aim of this systematic review will be to assess the effectiveness of approaches for the primary prevention of food allergy.

**Methods:**

Seven bibliographic databases will be searched from their inception to September 30, 2012 for systematic reviews, randomized controlled trials, quasi-randomized controlled trials, controlled clinical trials, controlled before-and-after studies, interrupted time series and cohort studies. Cohort studies will be included due to an inability to randomize with interventions such as breastfeeding. Studies that focused on the development of either food sensitization (a proxy measure) or food allergy will also be eligible for inclusion. Studies will be critically appraised using the Critical Appraisal Skills Program and Cochrane Risk of Bias tools, as appropriate.

**Discussion:**

There is a lack of rigorous evidence to support recommendations about how to prevent the development of food allergy. It would appear that it is important to see the prevention of food allergy in the context of individual, family and wider factors that may influence its development. There is much left to learn about preventing food allergy, and this is a priority given the high societal and healthcare costs involved. This systematic review will help to further this learning.

## Background

Allergies to foods such as milk, eggs, peanuts and tree nuts can have a significant effect on people’s quality of life and physical functioning, and can also be costly in terms of medical visits and treatments [1]. Given the morbidity resulting from food allergy, there is considerable scientific, professional and lay interest in approaches that may reduce the risk of individuals developing food allergy. A wide range of antenatal, perinatal, neonatal and childhood interventions have been investigated, and it is therefore important that data about the effectiveness and safety of these primary prevention strategies is assessed and synthesised.

The European Academy of Allergy and Clinical Immunology (EAACI) is developing guidelines about how to prevent and manage food allergy. As part of the guidelines development process, a systematic review is planned to examine published research about the prevention of food allergy. This systematic review is one of seven inter-linked evidence syntheses that are being undertaken in order to provide a state-of-the-art synopsis of the current evidence base in relation to epidemiology, prevention, diagnosis and clinical management, and impact on quality of life, which will be used to inform clinical recommendations.

### Aims

The aim of this systematic review will be to assess the effectiveness of approaches for the primary prevention of food allergy.

### Scope

The umbrella term ‘food hypersensitivity’ is used to describe any adverse reaction to food [2]. The term ‘food allergy’ refers to the subgroup of food-triggered reactions in which immunological mechanisms have been implicated, whether IgE-mediated, non-IgE-mediated, or involving a combination of IgE- and non-IgE-mediated etiologies [3]. All other reactions to food that have sometimes been referred to as ‘food intolerance’ constitute non-allergic food hypersensitivity reactions and are outside the focus of this enquiry.

The topic of food allergy is complicated by the fact that IgE-mediated reactions can manifest as angioedema, urticaria, atopic eczema/dermatitis, oral allergy syndrome and anaphylaxis, for example. Non-IgE-mediated immunological reactions result from activation of other immunological pathways (e.g. T-cell mediated) and can manifest as atopic eczema/dermatitis, gastro-esophageal reflux disease, food protein-induced enterocolitis, proctocolitis, and enteropathy syndromes. The contemporary definition of food allergy thus includes several clinical entities with different pathophysiologies resulting from exposure to different foods [4]. For simplicity, the review will only examine studies that seek to prevent food allergy or food sensitivity as a primary or secondary outcome. Studies seeking to prevent other manifestations such as eczema will not be included. Coeliac disease is an important non-IgE mediated condition but as it has distinct symptoms and prognosis different from atopic conditions it will be excluded from this review [4et].

## Methods

### Inclusion criteria

**Figure 1:**
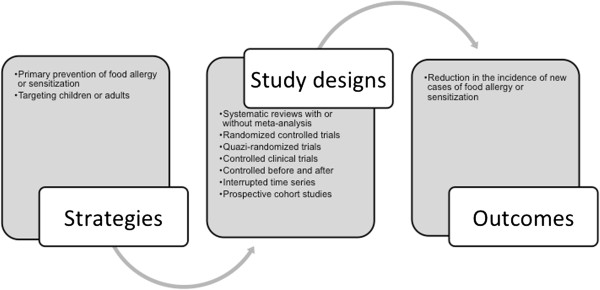
Conceptualisation of systematic review on the prevention of food allergy.

We have conceptualised the review to incorporate the interventions, study designs and outcomes, as shown in Figure 1: Conceptualisation of systematic review on the prevention of food allergy.

Study designs eligible for inclusion in the review comprise:

•systematic reviews with or without meta-analyses

•randomised controlled trials

•quazi-randomised controlled trials and controlled clinical trials (defined as studies where the comparison group is not fully randomised)

•controlled before and after studies (only where a clearly defined comparison group is available prospectively) and interrupted time series studies (where measures are taken during at least three time points before and at least three time points after intervention)

•prospective cohort studies.

We suspect that there will be limited information available from systematic reviews and randomised trials, so we have opted to include lower forms of evidence where non-random allocation of patients has occurred [5]. Prospective cohort studies, despite being lower forms of evidence, will be included as advice from experts at EAACI suggests that studies looking at breastfeeding and its role in preventing food allergy will be missed by the other study designs.

Studies already included in other systematic reviews will be eligible for quality appraisal and inclusion in this review. Where repeated reports of the same study are identified, the most up-to-date or detailed will be included unless there is a good clinical reason to include earlier studies.

Only research published as full papers will be eligible for inclusion.

### Exclusion criteria

The following material will be excluded from the review:

•non-systematic reviews, discussion papers, non-research letters and editorials

•qualitative studies

•case studies, case series, non-controlled before and after studies, and other lower quality designs

•animal studies

•abstracts and studies not available in full form

•unpublished material

•studies about risk factors (as these are covered in another review in the series).

### Search strategy

We will search the following databases:

•Cochrane Library, including:

oCochrane Database of Systematic Reviews (CDSR)

oDatabase of Reviews of Effectiveness (DARE)

oCENTRAL (Trials)

oMethods Studies

oHealth Technology Assessments (HTA)

oEconomic Evaluations Database (EED)

•MEDLINE (OVID)

•Embase(OVID)

•CINAHL (Ebscohost)

•ISI Web of Science (Thomson Web of Knowledge)

•TRIP Database (http://www.tripdatabase.com)

•Clinicaltrials.gov (NIH web)

A highly sensitive search strategy has been developed and validated study design filters will be applied to retrieve relevant articles. To retrieve systematic reviews, we will use the systematic review filter developed at McMaster University Health Information Research Unit (HIRU). To retrieve randomised controlled trials (RCTs), we will apply the Cochrane strategy for identifying trials in MEDLINE: sensitivity- and precision-maximising version (2008 revision); Ovid format from Chapter 6 of the Cochrane Handbook [6]. To retrieve non-randomised studies, i.e. controlled clinical trials (CCT), controlled before-and-after (CBA), and interrupted time-series (ITS) studies, we will use the Cochrane Effective Practice and Organisation of Care (EPOC) filter Version 2.4, available on request from the EPOC Group [7,8]. Cohort studies will be retrieved using methods that have been developed in the context of other systematic reviews.

The search strategy has been devised on OVID MEDLINE and then adapted for the other databases (see Additional file 1 for full search strategies). In all cases the databases will be searched from inception to 30 September 2012. Additional references will be located through searching the references cited in systematic reviews and through discussion with experts in the field. We will invite experts who are active in the field from a range of disciplines and locations to comment on our search strategy and the list of included studies. No language restrictions will be applied and, where possible, all literature will be translated. Additional studies will be sourced through experts in the field and hand-searching up until 31 December 2012. All references will be imported into an EndNote Library and tagged with the name of the database.

### Study selection

The titles of identified studies will be checked independently by two reviewers according to the above selection criteria, and categorised as: included, not included and unsure. For those papers in the unsure category, we will retrieve the abstract and re-categorise as above. Any discrepancies will be resolved by consensus and, if necessary, a third reviewer will be consulted. Full text copies of potentially relevant studies will be obtained and their eligibility for inclusion independently assessed. Studies that do not fulfil all of the inclusion criteria will be excluded.

### Quality assessment strategy

Quality assessments will independently be carried out on each study by two reviewers using the relevant version of the Critical Appraisal Skills Programme (CASP) quality assessment tool for systematic reviews [9]. We will assess the risk of bias using the criteria suggested by EPOC [10]. RCTs, CCTs and CBAs will be assessed for generation of allocation sequence, concealment of allocation, baseline outcome measurements, baseline characteristics, incomplete outcome data, blinding of outcome assessor, protection against contamination, selective outcome reporting and other risks of bias. For ITS designs we will also assess the independence of the intervention from other changes, the pre-specified shape of the intervention and if the intervention was unlikely to affect data collection. These assessments will draw on the principles incorporated into the Cochrane EPOC guidelines for assessing intervention studies [11] and the Strengthening the Reporting of Observational Studies in Epidemiology for assessing observational studies [12]. Any discrepancies will be resolved by discussion or, if agreement cannot be reached, by arbitration by a third reviewer.

### Analysis, data synthesis and reporting

Data will be independently extracted onto a customised data extraction sheet by two reviewers, and any discrepancies will be resolved by discussion or, if agreement cannot be reached, by arbitration by a third reviewer. A descriptive summary with data tables will be produced to summarise the literature. The focus will be a narrative synthesis. If clinically and statistically appropriate, meta-analysis using either fixed-effect or random-effects modelling will be undertaken using methods suggested by Agresti and Coul [13].

This review has been registered with the International Prospective Register of Systematic Reviews (PROSPERO) and has registration number CRD42013003709 allocated to it. The Preferred Reporting Items for Systematic Reviews and Meta-Analyses (PRISMA) checklist will be used to guide the reporting of the systematic review [14].

## Discussion

To date, there exists a lack of rigorous evidence to support recommendations about how to prevent the development of food allergy. There is some evidence to support breastfeeding and partially or extensively hydrolyzed whey and casein formula to prevent food allergy, especially in infants at high-risk. However, there is little evidence to support recommending changes to the diets of pregnant or lactating mothers, supplements for mothers or infants or delaying the introduction of solid foods as a way of protecting against food allergy. Multifaceted interventions that reduce exposure to both food and environmental allergens may be worth further exploration.

It would appear that it is important to see the prevention of food allergy in the context of individual, family and wider factors that may influence its development. Large trials are ongoing to examine some components, such as the timing of introducing potentially allergenic foods. Education interventions and strategies that target the determinants of food allergy, especially in those at high-risk, may also be worth investigating further using high quality designs. There is much left to learn about preventing food allergy, and this is a priority given the high societal and healthcare costs involved. We believe that this systematic review will help to further the learning and identify gaps that need to be filled through future research endeavours.

## Abbreviations

CASP: Critical appraisal skills programme tool; CBA: Controlled before-and-after studies; CCT: Controlled clinical trials; CDSR: Cochrane database of systematic reviews; DARE: Database of reviews of effectiveness; EAACI: European academy of allergy and clinical immunology; EED: Economic evaluations database; EPOC: Effective practice and organisation of care; HTA: Health technology assessments; ITS: Interrupted time-series studies; PROSPERO: Prospective register of systematic reviews; PRISMA: Preferred reporting Items for systematic reviews and meta-analyses; RCT: Randomised controlled trials

## Competing interests

The authors declare that they have no competing interests.

## Authors’ contribution

DdeS, SSP, ST and TR conceptualised and designed the protocol and drafted earlier versions of the document in their capacity as methodologists. SH and AM contributed to further refinements of the protocol and revised it critically for important intellectual content in their capacity as guideline leads. AS led on the development of concepts used in this protocol and revised it critically for important intellectual content in his capacity as the methodology lead. All authors approved the final version to be published.

## Supplementary Material

Additional file 1 Search strategies. Click here for file
